# Evaluation of the Elemental Composition of Dietary Supplements Containing Iron Available on the Polish Market Using ICP-OES, FAAS and CVAAS Techniques

**DOI:** 10.3390/molecules30234511

**Published:** 2025-11-22

**Authors:** Elżbieta Maćkiewicz, Martyna Klimaszewska, Jadwiga Albińska, Małgorzata Iwona Szynkowska-Jóźwik

**Affiliations:** Institute of General and Ecological Chemistry, Faculty of Chemistry, Lodz University of Technology, Zeromskiego 114, 90-543 Lodz, Polandmalgorzata.szynkowska@p.lodz.pl (M.I.S.-J.)

**Keywords:** dietary supplements, iron, elemental composition, ICP-OES, FAAS, CVAAS

## Abstract

The Polish dietary supplement market is undergoing rapid development, driven by an increasing societal interest in preventative healthcare and the utilisation of products that have demonstrated efficacy. Poland is a leading European dietary supplement market, driven by a number of factors, including increased nutritional awareness, an ageing population, and intensive marketing efforts by manufacturers. In this study, the elemental composition of 24 dietary supplements containing iron, folic acid, and other vitamins and essential macro- and micronutrients was examined using CV AAS, F AAS, and ICP-OES techniques. The samples analysed included supplements intended for pregnant and breastfeeding women, supplements for individuals struggling with anaemia, and multivitamin supplements containing a complete set of both vitamins and minerals. In order to ensure the accuracy of the product, the mineral doses listed in the package inserts were also verified. The study’s findings revealed significant discrepancies between the doses declared by manufacturers and the doses obtained through analysis, particularly with regard to iron and zinc. Furthermore, an inconsistency was observed between the mineral doses and the values recommended by Polish law.

## 1. Introduction

In recent years, there has been a marked increase in the popularity of dietary supplements containing iron and folic acid. The demand for these preparations is growing among women who are planning a pregnancy, who are pregnant, or breastfeeding, as well as those whose primary goal of supplementation is to support a healthy appearance. In both the European Union and the United States, dietary supplements are recognised as foodstuffs that allow for supplementation of a normal diet with ingredients that provide beneficial nutritional and/or physiological benefits [1,2]. In accordance with the provisions stipulated in Directive 2002/46/EC of the European Parliament and of the Council of 10 June 2002, these substances encompass vitamins, minerals, and other ingredients that exert a physiological effect. The presence of such substances in dietary supplements necessitates additional assessments of safety and compliance with national law [2,3,4]. However, the Dietary Supplement Health and Education Act of 1994 (DSHEA), in force in the United States, indicates that, in addition to vitamins and minerals, a dietary supplement may contain plant ingredients, concentrates, metabolites, amino acids, and other substances intended to increase dietary reference values [1]. In its Global Report on Traditional and Complementary Medicine 2019, the WHO highlights the absence of coherent and sufficiently rigorous mechanisms for the supervision of dietary supplements on a global scale [5]. In the European Union, the preparation of such products is subject to the requirement of Good Manufacturing Practices, and the onus is on each manufacturer to ensure that the finished product is in compliance with the relevant regulatory framework. The introduction of dietary supplements to the US market is less complex and less restrictive than in the European Union, as registration and verification of finished products by the Food and Drug Administration is not a prerequisite [1]. In accordance with the aforementioned legislative acts, dietary supplements are required to be presented in a form that facilitates straightforward dosing. This stipulation signifies that they can be introduced to the market in forms analogous to those of medicinal products [1,6]. The similarity in form and packaging of dietary supplements has led to a perception that they are as effective and safe as pharmaceuticals. Moreover, the ease with which dietary supplements can be introduced to the market, in conjunction with the paucity of clinical trials, enables manufacturers to do so with alacrity. The chemical forms of dietary supplement ingredients produced in the EU are subject to compliance with applicable regulations, given their pivotal role in bioavailability and efficacy [7]. Dietary iron occurs in two forms: heme and non-heme forms (highly bioavailable Fe^2+^ and low-bioavailable Fe^3+^) [8]. The mechanism of absorption of this microelement by enterocytes differs between heme and non-heme iron [9]. Trivalent iron is first reduced to the divalent form, which is then transported into enterocytes by DMT1 [10,11]. In contrast, heme iron is absorbed as an intact molecule through the HCP1 transporter, resulting in higher bioavailability compared with non-heme iron [11,12]. Heme iron is also less affected by absorption inhibitors such as phytates and polyphenols [8,13].

The predominant forms of iron present in dietary supplements are ferrous fumarate and ferrous bisglycinate, with varying concentrations of elemental iron [7]. Dietary supplements containing divalent iron are absorbed four times more efficiently than those containing trivalent iron [14]. This relationship is attributable to the poor solubility of Fe(III) in the alkaline environment of the small intestine. A notable disadvantage associated with oral iron(II) salts is the occurrence of various adverse gastrointestinal effects [15]. Excessive intake of iron, particularly when combined with a diet abundant in this element and the regular use of supplements exceeding recommended guidelines, can result in its accumulation within the body. However, iron dosages below the established standards do not meet the daily requirement, thereby preventing effective erythropoiesis and the prevention of anaemia [16].

The issue of the quality of dietary supplements is becoming increasingly common. Puścion-Jakubik et al. utilised flame atomic absorption spectrometry to ascertain iron concentrations in 109 dietary supplements procured on the Polish market between 2022 and 2023 [17]. The researchers then drew parallels between the findings and the information declared on the labels. The determined iron content was consistent with the manufacturer’s declaration in only 19.27% of the tested dietary supplements. Over the course of the study, it was found that 11% of the samples had an Fe content that was lower than that declared on the packaging, while 69.73% had higher iron content [17]. Research has indicated that the iron content determined by the F AAS method in dietary supplements frequently exceeds the declared value. The study found that 52% of the tested preparations available in Poland had a higher Fe content than declared on the packaging, while the remaining 48% of samples had a lower content [18]. Furthermore, disparities in iron content were identified between chemical forms of iron salts and pharmaceutical forms of finished dietary supplements [17]. Furthermore, Surowiecka et al. highlighted that in the majority of the examined preparations, the dosage stipulated by the manufacturers did not attain the recommended daily allowance (RDA). This is of particular significance in sensitive groups, such as pregnant women [18].

The present study examined a total of 24 dietary supplements containing iron, folic acid, and vitamin C, with some samples also containing other vitamins and minerals. The objective of the present study was to evaluate the safety of these dietary supplements with respect to their mineral content, including Fe, Ca, Cr, Cu, Mg, Mn, Mo, and Zn, as well as their heavy metal content, i.e., Cd, Hg, and Pb. The objective was achieved by measuring elemental ion concentrations using ICP-OES and F AAS techniques, and total mercury content using CV AAS.

## 2. Results and Discussion

### 2.1. ICP-OES, AAS and CVAAS Results

Multi-element analysis of the studied dietary supplements was performed using ICP-OES. In addition to the most interesting mineral component, iron, the content of a number of other elements was analysed, including other microelements, macroelements, toxic elements, and heavy metals. Given the finding that the mineral composition of certain dietary supplement samples, notably those formulated for pregnant women, was found to be particularly abundant, it was deemed essential to verify the content of specific analytes by means of an alternative analytical technique, namely FAAS. It was determined that high iron concentrations, present in certain samples, resulted in spectral interferences in the ICP-OES technique [19,20]. Consequently, further analyses were conducted using F AAS for elements such as Fe, Pb and Zn.

#### 2.1.1. Characteristics of the Elemental Composition of the Studied Dietary Supplements

The results of the analyses performed regarding the elemental composition were collated and statistically analysed, thus providing the mean, minimum, maximum, median and standard deviation values. The results of this study are presented in Table 1. Iron is present in dietary supplements in a variety of forms. At present, the most prevalent compounds are iron(II) bisglycinate or fumarate (20 samples). In the remaining cases, the active ingredient was either unspecified chelate, gluconate, or pyrophosphate, or the name of the active ingredient was not included on the product leaflet.

Iron, the element most extensively analysed in the dietary supplements under scrutiny, is a critical microelement for human health. According to the labelling, some of the supplement products that were tested contained high iron content and should only be used by individuals with increased iron requirements. The analysis results indicate that dietary supplement sample no. 21 exhibited the lowest iron content, with a value of 2993 mg/kg. This dietary supplement, formulated for pregnant and breastfeeding women, is notable for its complexity of composition. The dietary supplement sample with the highest iron concentration was identified as sample 13. The product known as 12 is intended for individuals with increased iron requirements and is registered on the US market. The median and mean iron concentration values obtained were 235.3 and 365.6 mg/kg, respectively (Table 1). This finding indicates statistically lower iron concentrations in the tested samples. This phenomenon can be attributed to the fact that the majority of the analysed products are designed exclusively to support the functioning of the human body and do not contain elevated concentrations of this element.

**Figure 1:**
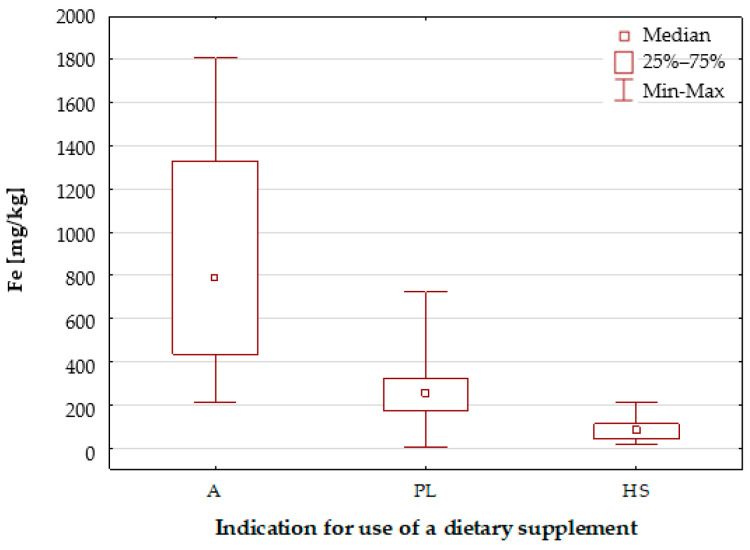
Box-and-whisker plot for Fe by indication for use of a dietary supplement: A— anaemia; PL—pregnancy & lactation; HS—hair & skin.

The dietary supplement samples that were analysed were divided into groups based on their intended use and composition (Figure 1, Table 2). An analysis of the division of the samples according to the intended use of the dietary supplement reveals that the highest median value was found in group A (A—anaemia; 789.4 mg/kg), which is for individuals with increased iron requirements (e.g., those struggling with anaemia). In contrast, the lowest median value was observed in the HS group (HS—hair & skin, 80.45 mg/kg), which is recommended for individuals seeking to maintain optimal health in their hair, skin, and nails. Concurrently, the A group demonstrates the greatest heterogeneity in terms of iron content, characterised by substantial variation in results between the minimum and maximum values. In contrast, the HS group demonstrates the highest level of homogeneity.

**Figure 2:**
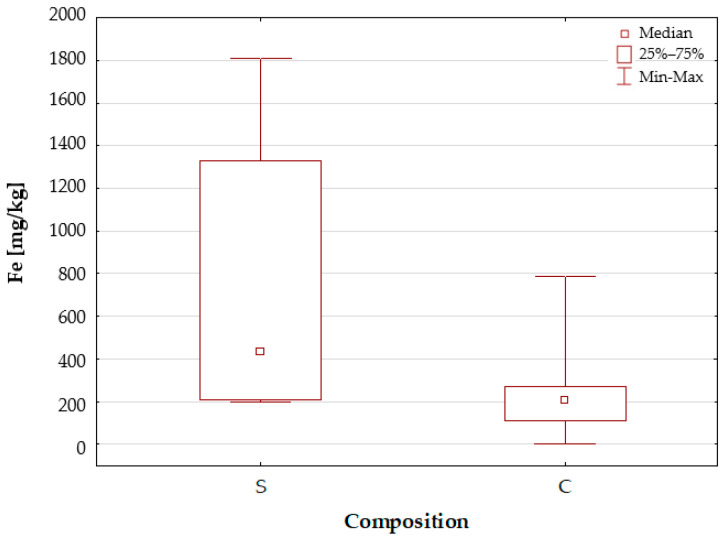
Box-and-whisker plot for Fe by composition of a dietary supplement: S—simple; C—complex.

Figure 2 presents a box-and-whisker plot for iron, utilising this categorisation to differentiate between samples based on their compositional complexity. For the group of dietary supplement samples with a simple composition (S), a wide range of results and a slightly higher median value of Fe concentrations were obtained compared to the group of samples with a complex composition, which, in addition to iron and folic acid, also contained a number of vitamins and other minerals. Furthermore, both graphs (Figure 1 and Figure 2) demonstrate results for samples characterised by extreme iron concentrations. It is evident that the samples exhibiting the lowest and highest concentrations bear a resemblance to the following samples: a dietary supplement (sample no. 21) characterised by a complex composition, which is intended for pregnant and breastfeeding women; and a dietary supplement with a complex composition, which is intended for individuals with increased iron requirements.

The subsequent section will present a detailed analysis of the composition and verification of the content obtained in the analyses. This will be undertaken with a view to establishing a correlation between the values declared on the packaging and the analysis contents.

For two macronutrients, calcium (Ca) and magnesium (Mg), concentrations were obtained that were significantly higher than the concentrations listed on the product labels. In the case of magnesium, this phenomenon can be attributed to the presence of additives in certain dietary supplements that have no significant effect on the body, in addition to active ingredients such as magnesium ascorbate. Examples of such additives include magnesium stearate, which functions as an anti-caking agent. The macroelements composition of the dietary supplements studied exhibited significant variation, attributable to the degree of variability in the samples. The samples studied included those containing primarily iron and folic acid, as well as those containing a full complex of vitamins and minerals, known as multivitamins. The minimum calcium content that was subjected to testing was 27.40 mg/kg, with this reference made to sample no. 9, a dietary supplement intended for individuals with increased iron requirements, which is a product with a simple composition. The maximum concentration that was ascertained was an astonishing 655,390 mg/kg, and this was related to sample no. 15. The product under discussion is a dietary supplement for pregnant women, and is labelled as such. As stated in the package insert, calcium carbonate was identified as the primary ingredient in the preparation. It is also noteworthy that the median calcium concentration value obtained was 4763 mg/kg, and the number of samples analysed with complex compositions significantly exceeded those with simple compositions.

Analogous results were obtained for another macronutrient—magnesium. The lowest recorded concentration was 12.36 mg/kg (sample no. 24, an effervescent dietary supplement with a rudimentary composition and no declared magnesium content), which was found to be the only sample with such a low magnesium concentration. Conversely, the highest magnesium concentration obtained was 270,720 mg/kg (sample no. 23, a dietary supplement containing a complex of vitamins and minerals). Notwithstanding the fact that magnesium oxide constitutes one of the primary ingredients of the tablet, the magnesium content of this sample, as declared on the product label, is comparatively low. Conversely, for the remaining macroelements, such as potassium, phosphorus and sulphur, considerably lower concentrations were obtained. This phenomenon can be attributed to the fact that these elements are not characteristic of dietary supplements, but rather constitute a component of the compounds contained therein. The analysis revealed that the supplement sample with the highest recorded potassium concentration was sample no. 16, which had a concentration of 2513 mg/kg. The highest phosphorus concentration was found in sample 22, a simple dietary supplement intended for pregnant women. The concentration of phosphorus in this dietary supplement was found to be 38,080 mg/kg, with phosphorus being the primary constituent of the filler, calcium phosphate. For the final macroelement analysed, sulphur, the highest content was recorded in sample no. 20, a complex dietary supplement for pregnant women. This concentration was found to be approximately 5%, which is notable given that this particular preparation comprised powdered bamboo shoots and exogenous amino acids. These substances are recognised as a substantial and readily obtainable source of sulphur for the human body. The content of macronutrients in the tested dietary supplements is therefore very diverse, resulting from the presence of active ingredients as well as from the presence of various types of compounds constituting fillers and compounds with anti-caking properties.

The analysis revealed a cobalt concentration of 39.89 mg/kg in a supplement sample, representing one of only two instances of high cobalt concentrations identified during the study. This supplement sample was accompanied by a package insert that indicated an unusually high content of vitamin B12, also known as cobalamin, with a dosage of 100 µg. This was sample no. 9, a dietary supplement characterised by a rudimentary composition, comprising the active ingredients ferrous fumarate, pteroylmonoglutamic acid, and vitamin B12.

For chromium, only three dietary supplements with complex compositions (samples 14, 17, and 23) declared the presence of chromium in the form of chromium picolinate or chromium (III) chloride (hexahydrate) according to the product information. The objective of these products was to promote a healthy appearance in women, encompassing the maintenance of healthy hair, skin, and nails. The chromium dose was approximately 25 µg, although one preparation contained as much as 40 µg of chromium. However, a significantly elevated result of 118.0 mg/kg was obtained for sample 17, while lower but nevertheless high concentrations of chromium were obtained for dietary supplement samples that did not contain declared chromium. As demonstrated in sample 9, the concentration of chromium was found to be 24.42 mg/kg.

The presence of copper was identified in the package inserts of seven of the 24 dietary supplements that were analysed. These supplements belonged to different categories. The highest concentration that has been documented was 2368 mg/kg (a complex dietary supplement for pregnant women, sample no. 19). The median Cu concentration was found to be a mere 0.528 mg/kg, indicating that in the majority of the samples analysed, the concentration of this analyte was low. It is noteworthy that one dietary supplement (sample no. 2, a complex dietary supplement intended for pregnant women) exhibited a copper concentration of 870.2 mg/kg, a value that was not disclosed in the package insert by the manufacturer.

Manganese, a crucial microelement, was detected in all of the dietary supplement samples analysed. The maximum concentration obtained was 2741 mg/kg for the dietary supplement sample (sample no. 13). It is interesting to note that, according to the product package inserts, manganese should only be present in six supplements. However, the results indicated that manganese concentrations were elevated in another six supplements (ranging from 165 to 1077 mg/kg), while in the remaining twelve samples, they were present at negligible levels, below 60 mg/kg. The elevated manganese levels observed in dietary supplements that do not disclose its presence on the product label are likely attributable to the incorporation of natural plant extracts, such as those derived from carrots, broccoli, or tomatoes (e.g., sample no. 12). However, it is imperative that such information is included on the packaging of such supplements, as failure to do so may result in an overdose if the consumer is unaware of this and also takes other vitamin preparations containing this element.

However, a notable exception is molybdenum, which is present in dietary supplements at trace levels, typically in the range of several dozen µg. Of the dietary supplements that were analysed, a mere five had their manufacturers declare the presence of molybdenum. For these supplements, elevated results were obtained, with a maximum concentration of 122.3 mg/kg (sample no. 17—a complex dietary supplement intended for women). In the remaining cases, minimal concentrations of the element were identified, with nine samples exhibiting concentrations that fell below the limit of detection.

Zinc, as well as manganese, was detected in all of the dietary supplement samples that were analysed. An analysis of the product leaflets revealed that only 15 supplements had zinc declared. The maximum concentration of this element, 226.8 mg/kg, was recorded for a sample of a women’s dietary supplement containing vitamins and minerals (complex sample no. 17). It is also worthy of note that one of the samples with undeclared zinc content exhibited a significant concentration of zinc, which once more raises concerns about the inadequacy of the control of the composition of dietary supplements available on the market and may lead to a potential overdose of this element.

The study also examined the presence of toxic elements and typical heavy metals, including cadmium (Cd), mercury (Hg), and lead (Pb). For aluminium, a single sample exhibited a notable aluminium content, with a measured value of 1205 mg/kg (sample no. 19—a complex dietary supplement intended for pregnant women). For the remaining samples, the concentrations obtained did not exceed 100 mg/kg. For cadmium, an extremely toxic heavy metal, four samples showed values above the limit of quantification, with a maximum concentration of 0.375 mg/kg. This concentration does not pose a risk to consumers and did not exceed the permissible limit for dietary supplements, which is 1.0 mg/kg in both Poland and the European Union [21,22]. In the case of mercury, another heavy metal, only two samples showed concentrations of 0.012 and 0.013 mg/kg. It is noteworthy that these dietary supplements were manufactured by the same company, with one formulation intended for women and the other specifically developed for pregnant and breastfeeding women. This finding suggests the potential for contamination of the raw materials or during the manufacturing process. Although the detected concentrations are low according to the applicable standards, the presence of mercury in dietary supplements is unequivocally prohibited [21,22]. In the majority of cases, the dietary supplements that were analysed were found to be free from lead. However, only four samples were found to have concentrations above the limit of quantification, with a maximum concentration of 2988 mg/kg (for sample 9, a simple dietary supplement intended for individuals with increased iron requirements). In accordance with the prevailing standards in Poland and the European Union, the maximum permissible concentration of lead in dietary supplements is 3 mg/kg. The analysis of the samples revealed that none of them exceeded this limit [21,22].

#### 2.1.2. Verification of the Composition of Dietary Supplements According to the Information Provided on the Product Labels

##### Iron

The Team for Diet Supplements at the Chief Sanitary Inspectorate has established the maximum permissible and safe doses of iron in a single tablet or capsule of a given dietary supplement, taking into account the average mineral content in the daily diet (these values apply to Polish legislation) [21]. Consequently, it was determined that dietary supplements registered on the Polish market are permitted to contain a maximum of 20 mg of iron per dose. This dosage is elevated for pregnant women, with a recommended intake of 30 mg. This data is included in Table 2. It should also be noted that, according to numerous studies, the Safe Level of Intake for adults, pregnancy and lactation is 40 mg [23]. For nutrients for which data are insufficient to establish a Tolerable Upper Intake Level (UL), the European Commission has requested that the European Food Safety Authority (EFSA) identify the highest level of intake for which there is reasonable certainty that no adverse effects will occur [24]. This is referred to as a safe intake level. Iron is an example of such an element. In contradistinction to ULs, the application of safe levels is more circumscribed: the consumption of amounts exceeding these levels does not inherently constitute a health risk, and these values cannot be utilised to estimate the proportion of the population that may be exposed to adverse effects.

As illustrated in Table 2, the manufacturer’s declared values, obtained during the analysis process, are presented for all the samples that were tested. In addition, the calculated percentage of the determined value compared to the declared value is also expressed as a percentage. Furthermore, the presence of iron oxides or hydroxides, which function as colour-imparting substances, has been identified in a number of the analysed products. This has the effect of significantly complicating the analysis of iron content. However, this information is of paramount importance and will be used and discussed during the analysis of the composition of individual dietary supplements (the second-to-last column of the table). The final column of Table 2 also provides information on whether the dose determined during the analysis is safe for the consumer (based on the doses determined by the Team for Diet Supplements). As demonstrated in Table 2, the minimum declared dose of Fe in the tested dietary supplements was 2.1 mg, with only 4 products having a dose below 10 mg (labelled value). The subsequent ten samples had a declared dose of approximately 14 mg of iron, and for the following nine supplements, the dose was 20 mg or more. Of the dietary supplements analysed, sample no. 13 (a dietary supplement registered on the US market) contained the highest and only such high dose of iron, namely 85 mg. Analysing the FAAS analysis results, it can be concluded that only two of the 24 dietary supplements tested had the iron content declared by the manufacturer (samples 2 and 7). Of particular note, samples 2, 3, and 4 represent the same dietary supplement intended for pregnant women. However, the packages of this product were purchased at different times and in different locations in order to verify the consistency of the supplement’s composition. A comprehensive analysis of the package inserts revealed that they originated from different production batches, with two of them being from two distinct production plants. Moreover, the manufacturer of this supplement asserts the presence of iron oxide as an excipient that imbues the final product with its colour. Consequently, sample 2, despite the presence of iron oxide, exhibits an iron content that is consistent with the declared value. This may suggest a deficiency in the active ingredient (iron(II) bisglycinate) or an insufficient quantity of iron as an excipient. The remaining two samples of the same dietary supplement (samples 3 and 4) have higher iron contents than declared, but the obtained results are different, reaching 28.83 and 25.68 mg, respectively. As illustrated by the example of this product, it can be deduced that the composition of dietary supplements is not sufficiently repeatable and should be subject to more frequent controls. Two dietary supplements contained significantly less iron than the value declared in the product leaflet. As indicated by the manufacturer, the composition of Sample 5 is expected to contain 28 milligrams of iron. However, the analysis yielded a measured content of 9.207 milligrams of this element. Moreover, as stated in the product leaflet, the substance under scrutiny contains iron oxide as a colouring agent. Notwithstanding this addition, the total iron content is considerably lower than declared. Sample no. 21, according to the manufacturer’s declaration, should contain 27 mg of iron, while the measured content is negligibly low, amounting to only 0.062 mg of iron per dose. Such a significant discrepancy between the declared and actual values may pose a risk to potential consumers of not receiving adequate iron despite supplementation. However, a cause for concern was identified in the remaining samples analysed, with 20 out of 24 exhibiting higher iron contents than declared. Approximately 30% of the tested samples exhibited higher iron concentrations than those declared. For instance, in sample no. 8, the declared iron concentration was 14 mg, while the measured dose was found to be 19.32 mg. This represents 138% of the manufacturer’s declared dose. Over 50% of the samples exhibited significant excesses in comparison to the declared values. For instance, sample no. 22, for which the package insert indicates an iron dose of 28 mg, exceeds the declared value. The measured iron content has been found to be as high as 51.07 mg, which is approximately 182% of the declared dose (sample contains iron oxide). It is also noteworthy to mention sample 13, for which the declared dose was considerably high, while the measured iron dose was almost double the declared amount (this sample contains iron-containing additives in the form of ferrous fumarate). Upon thorough examination of the calculated percentage values, it is evident that sample 14 exhibited the most significant excess, which is approximately 214% of the declared dose. The Safe Level of Intake for iron is 40 mg. Five of the dietary supplements tested exceeded this value, including one significantly, sample 22, which obtained a single dose of 51.07 mg.

**Table 2:** Characteristics of the tested dietary supplements, comparison of the Fe content given in the leaflet with the content determined in the FAAS analysis (composition: S—Simple; C—Complex), Intended use: A—anaemia; HS—hair & skin; PL—pregnancy & lactation).

**Daily Intake Value** [mg], [21]	**Safe Level of Intake** [mg], [23,24]	**Composition**	**Intended Use**	**Sample No.**	**Labelled Value** [mg]	**Determined Value** [mg]	**Percentage of Determined Value Versus Labelled Value** [%]	**Iron as an Excipient**	**Safe Dose** [21]
*20 **30	40	S	A	1	30.00	44.73	149.1	No	No
		C	PL	2	14.00	14.69	104.9	Yes	Yes
		C	PL	3	14.00	28.83	205.9	Yes	Yes
		C	PL	4	14.00	25.68	183.4	Yes	Yes
		S	A	5	28.00	9.207	32.88	Yes	No
		S	PL	6	13.50	20.68	153.2	No	No
		C	PL	7	14.00	15.32	109.4	No	Yes
		S	PL	8	14.00	19.32	138.0	No	Yes
		S	A	9	20.00	41.71	208.5	No	No
		C	PL	10	10.00	13.00	130.0	Yes	Yes
		C	PL	11	14.00	29.24	208.9	No	Yes
		S	A	12 (U.S.)	25.00	38.61	154.4	No	No
		C	A	13 (U.S.)	85.00	159.2	187.3	Yes	No
		C	HS	14	14.00	30.07	214.8	No	No
		C	PL	15	20.00	34.91	174.5	Yes	No
		C	HS	16	7.000	9.363	133.8	Yes	Yes
		C	HS	17	3.220	4.699	145.9	Yes	Yes
		C	PL	18	28.00	40.95	146.2	No	No
		C	PL	19	17.00	26.57	156.3	No	No
		C	PL	20	14.00	21.84	156.0	No	Yes
		C	PL	21	27.00	0.062	0.230	No	No
		S	PL	22	28.00	51.07	182.4	Yes	No
		C	HS	23	2.100	3.313	157.8	Yes	Yes
		C	HS	24	7.000	9.377	134.0	No	Yes

*—In the case of dietary supplements specifically formulated for individuals with elevated iron requirements, **—In the case of dietary supplements intended for pregnant women.

**Figure 3:**
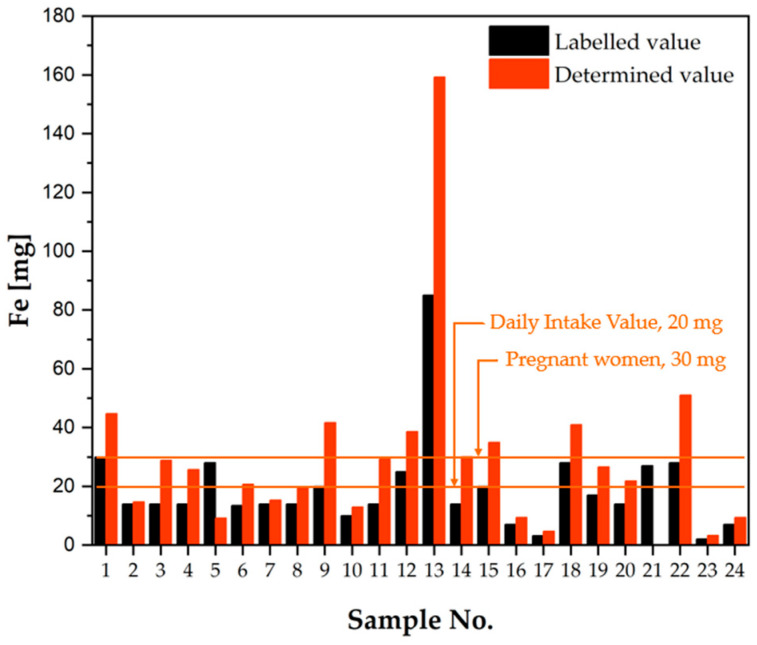
Comparison of the iron content declared in dietary supplement leaflets with the values determined in the analysis [mg/dose].

In order to provide a more accurate illustration of the manufacturer’s declared iron content relative to the labelled content, this information has been combined and presented in Figure 3.

##### Other Minerals

In addition to the primary component under scrutiny, i.e., iron, the content of other mineral compounds enumerated in the information leaflets accompanying dietary supplement packaging was also analysed and compared. The contents of the aforementioned documents are summarised and presented in Table 3 and Table 4.

In the context of calcium, it was observed that five of the dietary supplements examined had a calcium content declaration in their respective leaflets (see Table 3). The analysis revealed that calcium levels were elevated in all of the samples analysed. This is attributable to the presence of excipients, colourants, and fillers such as dicalcium phosphate and calcium carbonate in addition to the active ingredients (samples 11, 13–15, and 18). In the case of the latter three, calcium carbonate constitutes the primary ingredient of the tablet, as it is listed first among the ingredients specified in the leaflet. In such cases, verification of the content of this ingredient is rendered entirely unfeasible. In the case of calcium, the Tolerable Upper Intake Level (UL) was set by the European Food Safety Authority (EFSA) at 2500 mg/day for adults, and thus, this limit was not exceeded for the dietary supplements that were tested in isolation. However, it should be noted that calcium is a macroelement, and therefore a component found in high concentrations in many food products, including water, such as highly mineralized water and dairy products. Conversely, a substantial body of research suggests that, in the contemporary context characterised by the fast pace of life, a significant proportion of the population experiences deficiencies in minerals and vitamins. This, in turn, results in a growing reliance on nutritional supplements. Secondly, the bioavailability of minerals and vitamins from dietary supplements is not as high as that from food [25,26,27]. Consequently, when supplementing, it is imperative to consider not only the content of individual minerals in the dietary supplement taken, but also all consumed foods and beverages.

For chromium, only three samples exhibited a declared presence of this element (Table 3). This element is a popular ingredient in vitamin and mineral preparations, but its dose is low, and according to the guidelines of the Team for Diet Supplements, the daily dose of chromium in supplements should not exceed 200 µg [21]. However, no Tolerable Upper Intake Level (UL) has been established for chromium (according to EFSA) [24,28]. 

**Table 3:** Results of Ca, Cr and Cu content determination in the tested dietary supplements: LV—labelled value (value placed on the dietary supplement label); DV—determined value (value obtained during analysis); DV vs. LV—percentage of determined value versus labelled value.

**No.**	**Ca** Daily Intake Value, 1500 mg [21] The Upper Tolerable Intake Level (UL), 2500 mg [24,29]					**Cr** Daily Intake Value, 0.200 mg [21]				**Cu** Daily Intake Value, 2 mg [21] The Upper Tolerable Intake Level (UL), 5 mg [24,30]			
	**LV** [mg]	**DV** [mg]	**DV vs. LV** [%]	**Safe Dose** [21]	**Ca as Other** **Ingredients**	**LV** [mg]	**DV** [mg]	**DV vs. LV** [%]	**Safe Dose** [21]	**LV** [mg]	**DV** [mg]	**DV vs. LV** [%]	**Safe Dose** [21]
11	150.0	223.3	148.8	Yes	Yes								
13	25.00	57.72	230.9	Yes	Yes					2.000	2.160	108.0	No
14	160.0	432.8	270.5	Yes	Yes	0.025	0.027	107.3	Yes				
15	240.0	432.8	180.3	Yes	Yes					1.000	0.963	96.26	Yes
16										0.500	0.630	126.0	Yes
17						0.026	0.040	155.0	Yes	0.230	0.322	140.1	Yes
18	200.0	786.5	353.3	Yes	Yes					1.000	1.002	100.2	Yes
19										1.000	1.239	123.9	Yes
20										0.500	0.672	134.4	Yes
23						0.040	0.048	120.5	Yes				

**Table 4:** Results of Mn, Mo, Mg and Zn content determination in the tested dietary supplements: LV—labelled value (value placed on the dietary supplement label); DV—determined value (value obtained during analysis); DV vs. LV—percentage of determined value versus labelled value.

**No.**	**Mg** Daily Intake Value, 400 mg [21] The Upper Tolerable Intake Level (UL), 250 mg [24,31]					**Mn** Daily Intake Value, 1.8 mg [21] Safe Level of Intake, 8 mg [24,32]				**Mo** Daily Intake Value, 0.350 mg [21] The Upper Tolerable Intake Level (UL), 0.6 mg [24,31]				**Zn** Daily Intake Value, 15 mg [21] The Upper Tolerable Intake Level (UL), 25 mg [24,31]			
	**LV** [mg]	**DV** [mg]	**DV vs. LV** [%]	**Safe Dose** [21]	**Mg as Other Ingredients**	**LV** [mg]	**DV** [mg]	**DV vs. LV** [%]	**Safe Dose** [21]	**LV** [mg]	**DV** [mg]	**DV vs. LV** [%]	**Safe Dose** [21]	**LV** [mg]	**DV** [mg]	**DV vs. LV** [%]	**Safe Dose** [21]
2	75.00	236.4	315.2	Yes	Yes									8.000	8.150	101.9	Yes
3	75.00	210.8	281.0	Yes	Yes									8.000	23.93	299.1	No
4	75.00	233.7	311.7	Yes	Yes									8.000	22.42	280.2	No
7														5.000	5.649	113.0	Yes
10														6.000	2.399	29.98	Yes
11														9.000	7.701	85.60	Yes
13						5.000	4.463	89.26	No					5.000	6.330	126.6	Yes
14	112.5	287.7	255.7	Yes	Yes	0.600	0.574	95.67	Yes	0.025	0.021	83.06	Yes	10.00	13.78	137.8	Yes
15						1.500	1.281	85.37	Yes	0.050	0.060	120.7	Yes	10.00	13.85	138.5	Yes
16						0.750	0.780	104.0	Yes	0.037	0.039	106.7	Yes	7.500	8.205	109.4	Yes
17										0.033	0.042	128.6	Yes	3.200	9.540	298.1	Yes
18	58.20	144.1	247.6	Yes	Yes	1.000	0.911	91.09	Yes					15.00	21.53	143.6	No
19														12.00	12.31	102.6	Yes
20														10.00	10.35	103.5	Yes
23	57.00	205.6	360.8	Yes	Yes	1.800	1.975	109.7	No	0.050	0.058	115.7	Yes	5.000	7.017	140.3	Yes

**Figure 4:**
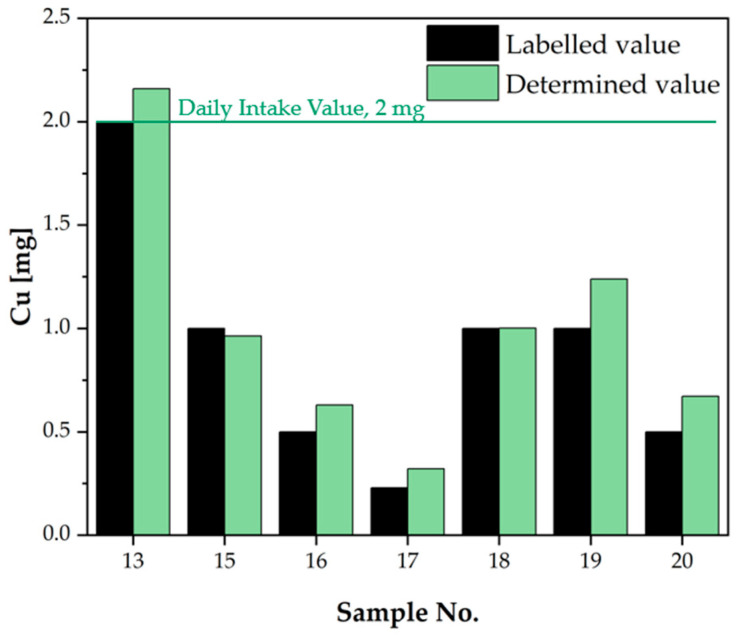
Comparison of copper content declared in dietary supplement leaflets with the values determined in the analysis [mg/dose].

Chromium (specifically trivalent chromium—Cr(III)) is a microelement considered to be relatively non-toxic in amounts naturally occurring in food. There have been no documented cases of Cr(III) toxicity in humans consuming it in the diet or supplements in amounts considered to be within the normal range. Nevertheless, the absence of an UL does not imply that chromium can be ingested without restrictions; it merely signifies that there is an absence of data to determine a safety limit. In the case of all three samples intended for use as vitamin and mineral preparations to strengthen hair, skin and nails (H,S), it was found that the obtained values exceeded the values declared on the product labels or were significantly higher. However, it is notable that none of the values examined exceeded the Daily Intake Value established by the Team for Diet Supplements [21]. Copper is a microelement that is present in a significant number of mineral dietary supplements (Table 3). The Team for Diet Supplement in Poland asserts that the maximum daily dose for adults should not exceed 2 mg [21]. According to the data provided by the European Food Safety Authority (EFSA), the maximum daily intake should not exceed 5 mg [30]. The established upper level of 5 mg/day is not applicable to pregnant or lactating women due to the inadequate data available for these life stages [31]. Of the 24 dietary supplements that were analysed, the copper content was detected in seven of them. As indicated on the product packaging, the presence of copper was observed in the form of amino acid chelates (gluconate, bisglycinate), oxide, or sulphate. In two cases, the leaflet failed to provide information regarding the copper compound that had been added to the supplement. In all cases, the values obtained were found to be close to or higher than the declared value. For sample 13, the declared content was 2 mg, while analysis revealed a slightly higher content of 2.160 mg. This finding is inconsistent with Polish regulations regarding the content of individual mineral compounds in dietary supplements [21]. The results obtained for copper were then compared and presented in Figure 4, with the Daily Intake Value indicated.

Magnesium is another macroelement for which Polish regulations define a maximum content of 400 mg in dietary supplements (Table 4) [21]. European regulations stipulate a maximum daily intake of 250 mg of magnesium for adults [31]. However, as with calcium, the magnesium content declared on product labels cannot be verified due to the presence of additives and fillers such as magnesium oxide, magnesium stearate, and talc. The magnesium content of the dietary supplements tested was found to be significantly higher, which can be attributed to the presence of additives.

**Figure 5:**
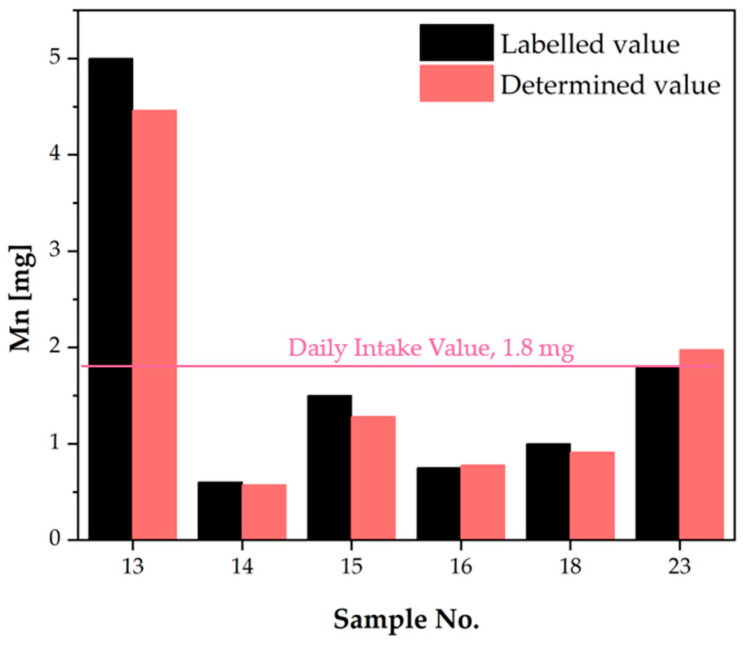
Comparison of manganese content declared in dietary supplement leaflets with the values determined in the analysis [mg/dose].

The analysis revealed that manganese was present in six of the examined supplements, with concentrations ranging from 0.6 to 5 milligrams per dose (Table 4). In accordance with the regulations established by the Polish authorities, the maximum permissible dosage of manganese in a dietary supplement is 1.8 mg [21]. The declared dose was exceeded in two dietary supplements: sample 13, for which the declared dose was 5 mg, but the analysis yielded 4.463 mg (this is a dietary supplement registered on the US market), and sample 23, for which the declared dose was 1.8 mg, but the analysis yielded 1.975 mg. For the remaining samples, the value obtained ranged between 85 and 104% of the declared value. It is also noteworthy that the Upper Tolerable Intake Level (UL) for manganese remains undetermined, with the Safe Level of Intake set at 8 mg [32]. The results obtained for manganese were then compared and presented in Figure 5, with the Daily Intake Value indicated.

The doses of the next microelement to be analysed, molybdenum, are minimal, ranging from 25 to 50 µg/dose (present in the five supplements tested as sodium molybdate (VI)). The doses obtained during the analysis (determined values) do not differ significantly from those declared, ranging from 83 to approximately 130% of the declared dose (Table 4). The stipulated dose of 0.35 mg, as outlined by Polish legislation, was not exceeded for any of the analysed supplements [21].

**Figure 6:**
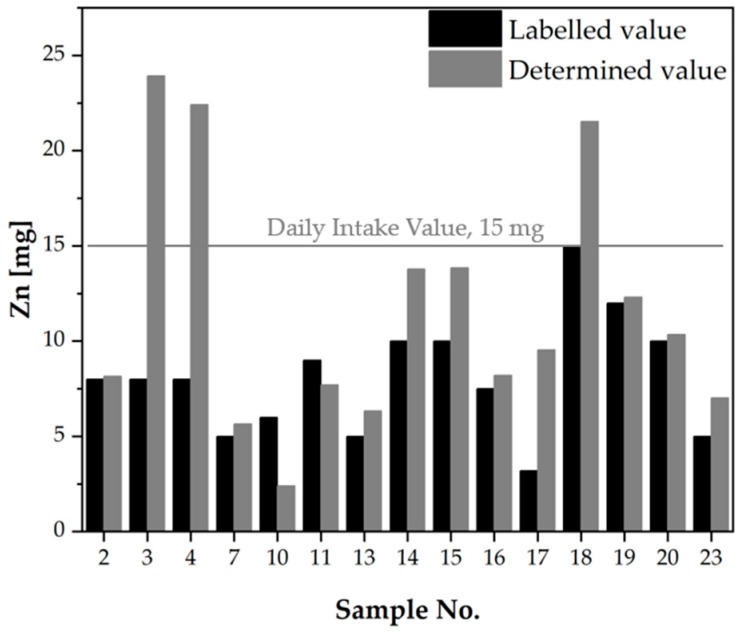
Comparison of zinc content declared in dietary supplement leaflets with the values determined in the analysis [mg/dose].

Zinc, the most prevalent mineral in dietary supplements, is a pivotal microelement that fulfils multiple functions in the body. The combination of zinc and iron in dietary supplements is a common practice, as these elements possess analogous metabolic functions and are both classified as micronutrients that are frequently lacking in individuals’ diets. It has been established that there are shared effects on the hematopoietic and immune systems. An essential element in this regard is iron, which is indispensable for the production of haemoglobin and the transport of oxygen. In addition, zinc is required for the functioning of hematopoietic enzymes and immunity, thereby complementing the effects of iron. Collectively, these elements contribute to the mitigation of anaemia and weakness [33]. In the case of zinc, only one sample yielded a dose that was significantly lower than the declared dose (sample no. 10–30% of the declared dose), while six samples yielded doses that were around 100% of the declared dose. A total of eight samples were found to contain values that were significantly higher than the declared dose, with some reaching up to three times the stated amount. For instance, samples 4, 17, and 3 yielded doses of 280, 298, and 299% of the declared dose, respectively. In accordance with Polish legislation, the maximum permissible dose of zinc in a dietary supplement is 15 mg [21]. However, the doses obtained in the analysis for samples 3, 4, and 18 significantly exceed this value (see Table 4). It is particularly noteworthy that some of the dietary supplement samples analysed are formulated for pregnant women, and the presence of mineral elements that approach the Upper Tolerable Intake Level (UL) is of significant concern. This may result in an oversupply of these elements, which could potentially lead to adverse effects. Furthermore, an analysis of the zinc results obtained for samples 2, 3 and 4 (samples of the same dietary supplement, originating from different production batches) revealed that the composition was not repeatable. Specifically, while the dose obtained for sample 2 was consistent with the declared value, significant excesses of this value were observed for samples 3 and 4. The results obtained for zinc were then compared and presented in Figure 6, with the Daily Intake Value indicated.

It is also important to emphasise that in Poland, the types of food additives are defined in the Act on Food and Nutrition Safety (implementing EU law, in particular, Regulation (EC) 1333/2008) [34]. The Act does not establish discrete “Polish” categories, but rather alludes to the functional groups of food additives that are enumerated in EU legislation. According to this regulation, dietary supplements primarily may include (i) colourants, i.e., substances intended to impart or restore colour, (ii) fillers, (iii) thickeners, (iv) glazing agents, and (v) antioxidants. Consequently, in accordance with prevailing regulations, the utilisation of food additives is not permitted for their nutritional properties. The primary function of these substances is to serve a specific technological purpose, such as preservation, colouring, stabilisation, thickening, or improving the texture of a product. Consequently, the majority of additives are introduced into food in minimal quantities, thus failing to contribute substantially to energy or nutritional value.

While certain groups of additives may possess secondary nutritional value, this attribute alone does not constitute a sufficient basis for their approval or utilisation. This encompasses modified starches or specific carriers capable of providing energy, reduced-energy polyols, and certain natural dyes, including carotenoids, which have been observed to exhibit provitamin A activity. In such instances, the nutritional properties are a consequence rather than an intended outcome of the technological process. Consequently, the nutritional value of these substances is negligible when compared to the active ingredients of dietary supplements.

## 3. Materials and Methods

### 3.1. Samples

The study examined the dietary supplement samples containing iron, folic acid and vitamin C, as well as formulations comprising an iron source, folic acid and a complex of vitamins and/or minerals procured from pharmacies, brick-and-mortar stores and online retailers, all operating within the Polish market. A variety of brands were represented among the samples, with selected dietary supplements traced to a common manufacturer. Samples were collected and tested in 2025. It is important to note that three samples of the same complex dietary supplement intended for pregnant women were procured for testing at different times. The three samples under scrutiny were obtained from disparate production batches. Two of the samples were obtained from a single production facility (samples 3 and 4), while the remaining sample was sourced from a second production facility. The samples in question were designated as samples 2, 3, and 4. Analysed supplements were available as capsules, including soft and hard capsules with conventional, rapid or modified release profiles, as well as coated tablets with conventional or extended release and one effervescent tablet. The dataset consisted of 24 dietary supplement samples, categorised according to intended use and composition. With regard to intended use, the products were divided into three categories: (A, *n* = 5) —formulations intended for individuals with increased iron requirements (e.g., for people with anaemia, athletes, women experiencing heavy menstrual bleeding); (PL, *n* = 14)—supplements designated for women planning pregnancy, during gestation or throughout lactation; (HS, *n* = 5)—formulations targeted at individuals with increased demand for vitamins and minerals, administered with the purpose of supporting a healthy appearance. In terms of composition, two categories were distinguished: simple formulations (S), containing iron together with a limited number of vitamins and complex formulations (C), where an iron source was combined with a broader range of vitamins and/or minerals. Most of these supplements, besides providing iron and folic acid, were commonly formulated with additional sources of Mg, Ca and Zn, together with various B-group vitamins. Whereas preparations for individuals with increased iron requirements more frequently appeared in the form of capsules containing iron and several vitamins (C, B_9_, B_12_). Dietary supplements used to support a healthy appearance (HS) mostly consisted of coated tablets containing a range of vitamins and minerals. In addition to serving as a source of iron, these preparations were frequently fortified with a range of microelements (Cr, Cu, Mn, Mo, Zn) as well as macroelements (Ca, Mg). Furthermore, beyond folic acid (vitamin B_9_), the complex formulations were additionally supplemented with vitamins C and E, together with B-group vitamins (B_1_, B_3_, B_5_, B_6_, B_12_). Additionally, two of the analysed formulations (sample 12, 13) were notified on the U.S. market, whereas the remaining products were registered in Poland. The division of the samples was achieved through analysis of the product composition in accordance with the information provided on their respective labels. A comprehensive analysis of the samples and the abbreviations employed in this study are both provided in Table 2.

### 3.2. Sample Preparation

Before analysis, tablets and the contents of hard capsules were finely ground in an agate mortar with a pestle to obtain a homogeneous powder. The powdered samples were weighed into test tubes using an analytical balance, with a sample mass of approximately 0.20 g. Next, 2 mL of 69% HNO_3_ (Suprapur, Merck, Darmstadt, Germany) and 0.5 mL of 36.5–38.0% HCl (J.T. Baker, Avantor, Radnor, USA) were added to each sample. Digestion was carried out using a closed-vessel, microwave-assisted system (UltraWave, Milestone, Milan, Italy). The wet mineralization process was performed in two successive steps (Table 5). The same digestion programme was applied to certified reference materials (CRMs) and blank samples. Since a considerable residue remained after the first digestion, each sample was supplemented with an additional 1 mL of 69% HNO_3_ (Suprapur, Merck, Darmstadt, Germany) and 0.5 mL of 36.5–38.0% HCl (J.T. Baker, Avantor, Radnor, PA, USA), and the digestion procedure was repeated to ensure complete decomposition.

Following mineralization, the obtained solutions were quantitatively transferred into volumetric flasks, and an internal standard (Yb, PlasmaCAL, Villebon sur Yvette, France) was added. The solutions were then diluted to a final volume of 25 mL and prepared in appropriate replicates. Samples that remained turbid after mineralization, forming suspensions, were centrifuged using an OHAUS centrifuge at 6000 rpm for 10 min to obtain clear supernatants suitable for subsequent analysis.

As no certified reference material with a comparable matrix was commercially available, materials with high iron content and a range of other trace elements were selected to verify the reliability and accuracy of the analytical procedure. The following reference materials were used: INCT-MPH-2, Mixed Polish Herbs (Institute of Nuclear Chemistry and Technology, Warsaw, Poland); 1570a, Trace Elements in Spinach Leaves (National Institute of Standards and Technology, Gaithersburg, MD, USA) and 1573a, Tomato Leaves (National Institute of Standards and Technology, Gaithersburg, MD, USA).

### 3.3. Instrumentation

#### 3.3.1. ICP-OES (Inductively Coupled Plasma–Optical Emission Spectrometry)

The elemental analysis was conducted using a Thermo Scientific™ dual-view ICP-OES spectrometer iCAP™ 7400 (Waltham, MA, USA). As illustrated in Table 6, the fundamental operational parameters of the ICP-OES spectrometer employed in the analysis are presented.

Prior to analysis, calibration solutions for the elements under investigation had to be prepared. For this purpose, a 100 mg/L CPAchem ICP multi-element standard solution (Stara Zagora, Bulgaria) and single-element standard solutions S (ICP grade, Analytika, Prague, Czech Republic) and P (ICP grade, PlasmaCAL, Courtaboeuf, France), each with a concentration of 1000 mg/L, were used. The range of the calibration curves was from the LOQ to the highest standard. The standard solutions were prepared by diluting the stock solutions with ultrapure water (Milli-Q, Milliopre, Bedford, MA, USA), which was used throughout the analysis.

Table 7 provides a comprehensive list of the analytical lines employed in the ICP-OES analysis. Table 7 provides a comprehensive list of the elements analysed, their respective analytical lines, limits of quantification, and the results obtained for the tested certified materials. The linear regression coefficient for each analyte ranged from 0.998 to 1.000. A total of three replicates were performed for each sample. Certified reference materials that had undergone prior mineralisation were prepared in duplicate with the objective of evaluating the accuracy of the proposed analytical procedure. This outcome substantiated the dependability of the sample preparation and the measurement protocol. Moreover, the employment of ytterbium as an internal standard facilitated uninterrupted regulation of the analytical signal during the course of the measurements.

#### 3.3.2. F AAS (Flame Atomic Absorption Spectrometry)

The determination of iron, zinc, and lead in dietary supplements was carried out using a High-Resolution Continuum Source Atomic Absorption Spectrometer HR-CS AAS (ContrAA 800 D, Analytik Jena, Jena, Germany), equipped with a flame atomization system.

Flame atomic absorption spectrometry (F AAS) is a competitive technique compared to other spectroscopic methods, particularly for samples in which the analyte concentration exceeds 50 μg/L. The relatively high iron content in the tested dietary supplements justified the use of flame atomization instead of inductively coupled plasma excitation (ICP-OES), where a significant level of spectral interferences may affect the accuracy of the measurements. The selection of the F AAS technique for the determination of Fe, Pb and Zn in dietary supplement samples was further justified by the fact that these elements exhibit highly selective absorption lines at 248.814, 217.000 and 213.857 nm, respectively (Table 8). The range of the calibration curves was from the LOQ to the highest standard.

Prior to analysis, the sample solutions were repeatedly diluted, which enabled the construction of calibration curves based on aqueous standard solutions of Fe, Zn and Pb. Absorbance measurements were performed in an acetylene–air flame at wavelengths corresponding to the high-efficiency working range of the diffraction gratings and CCD detector. These analytical conditions minimised the risk of spectral interferences, ensured greater signal stability, calibration curve linearity and enhanced the accuracy and reliability of the determinations. Each absorbance measurement was carried out in triplicate to further increase the robustness of the results. The analytical results for the CRM solutions showed good agreement with the certified concentrations, with recoveries approaching quantitative levels (Table 9).

#### 3.3.3. CV AAS

The Cold Vapour Atomic Absorption Spectroscopy (CV AAS) technique was employed for the determination of total mercury in dietary supplement samples. The direct mercury analyser Mercury MA-3000 (Nippon Instruments Corporation, Tokyo, Japan) does not require a dedicated sample preparation step. Approximately 50 mg of the homogenised material was weighed into ceramic boats. The ceramic boats had been previously preheated at high temperature and were stored in a desiccator prior to analysis. The weighed samples were then placed in the analyser. The range of the calibration curves was from the LOQ to the highest standard. The limit of quantification was determined to be 0.004 mg/kg. The following results were obtained for the studied reference materials: INCT-MPH-2, the declared value was 17.6 ± 1.6 µg/kg and the obtained values were (x_mean_ ± SD) 18.0 ± 1.1 µg/kg; 1570a, the declared value was 29.7 ± 2.1 µg/kg and the obtained values were (x_mean_ ± SD) 28.5 ± 2.0 µg/kg; 1573a, the declared value was 34.1 ± 1.5 µg/kg and the obtained values were (x_mean_ ± SD) 33.8 ± 1.2 µg/kg. The operating parameters of the MA-3000 mercury analyser are presented in Table 10.

### 3.4. Data Analysis

The statistical and multivariate analyses were performed using Statistica 12.5 (New York, NY, USA) software.

## 4. Conclusions

An analysis of the Polish dietary supplements market reveals constant and dynamic development, driven by two key factors. Firstly, there is growing health awareness amongst consumers, and secondly, there are intensive marketing activities by producers. The analyses performed indicate that a total of nine samples meet the safety requirements specified in Polish legislation (the Team for Diet Supplements). These samples are characterised by their high mineral content, with the mineral content of all tested preparations representing 37.5% of the total. However, 15 samples did not meet the safety requirements for iron, zinc, copper, and manganese, which collectively account for 62.5% of the tested dietary supplements. The preparations included eight formulations intended for women experiencing or planning a pregnancy, those who are pregnant, and/or breastfeeding; five formulations intended for individuals with increased iron requirements (e.g., anaemia); and two samples used to support healthy hair, skin, and nails. The findings of the present study suggest the necessity for additional research to be conducted into the actual effectiveness and safety of supplements, particularly in the context of their widespread availability and limited oversight of product quality. Insufficient control is still observed at two distinct stages: firstly, when supplements are introduced to the market, and secondly, when their composition is monitored post-market introduction. This may lead to discrepancies between the declared and actual effects of the preparations. A further significant problem is the low level of public awareness regarding the differences between dietary supplements and medicinal products. Many consumers mistakenly conflate these categories, attributing therapeutic benefits to them. The analysis results suggest the necessity to strengthen public health education so that decisions regarding supplementation are based on sound scientific knowledge, rather than solely on advertising messages. It is vital that, in the long term, a balance is achieved between the development of the supplement market, the effective institutional oversight thereof, and the protection of public health. This should be a key element of health policy in Poland.

## Figures and Tables

**Table 1:** Contents of elements in the measured dietary supplement samples (*n* = 24) [mg/kg].

Element	Mean	Median	Minimum	Maximum	Std. Dev.
Al	84.84	32.96	3.457	1205	239.6
Ba	1.456	0.853	0.180	6.006	1.359
Ca	76,080	4763	27.40	655,390	163,290
Cd	0.031	<LOQ	<LOQ	0.375	0.083
Co	4.373	1.545	0.158	39.89	8.757
Cr	12.54	3.545	<LOQ	118.0	26.25
Cu	365.5	0.528	<LOQ	2368	609.2
Fe	365.6	235.3	2.993	1810	423.0
Hg	0.003	0.002	<LOQ	0.013	0.003
K	351.0	94.99	<LOQ	2513	641.7
Mg	59,310	350.3	12.36	270,720	106,940
Mn	456.5	110.3	0.697	2741	762.9
Mo	13.01	0.185	<LOQ	122.3	30.32
P	5535	307.5	13.80	38,080	10,520
Pb	0.237	<LOQ	<LOQ	2.988	0.682
S	7621	1721	75.19	49,780	13,660
Sr	7.910	1.814	<LOQ	43.49	12.71
Zn	86.51	73.23	2.161	226.8	72.96

LOQ—limit of quantification.

**Table 5:** Microwave digestion process conditions.

**Stage**	**Time** [min]	**E** [W]	**T_1_** [°C]	**T_2_** [°C]	**P** [bar]
1	10	1500	220	70	150
2	20	1500	220	70	150

**Table 6:** Instrumental conditions and operating parameters for ICP-OES.

Instrument Parameter	Operating Conditions
Generator power [W]	1150
Carrier gas	Argon
Plasma gas flow rate [L/min]	12
Auxiliary gas flow rate [L/min]	0.5
Nebulizer gas flow rate [L/min]	0.5
Nebulizer	Concentric quartz
Torch	Quartz

**Table 7:** Limits of quantification (LOQ) for the proposed analytical procedure for dietary supplement analysis and results obtained for the studied certified materials using ICP-OES [mg/kg].

**Analyte**	**LOQ**	**INCT-MPH-2** Certified	**INCT-MPH-2** Obtained x_mean_ ± SD	**1570a** Certified	**1570a** Obtained x_mean_ ± SD	**1573a** Certified	**1573a** Obtained x_mean_ ± SD
Al 396.152 ^a^	0.031	670 ± 111	681 ± 15	310 ± 15	300 ± 21	598 ± 7.1	602 ± 4.9
Ba 493.409 ^a^	0.020	32.5 ± 2.5	31.8 ± 0.9	-	-	*63*	59 ± 0.9
Ca 393.366 ^r^ (%)	0.042	1.08 ± 0.07	1.01 ± 0.02	1.53 ± 0.07	1.50 ± 0.02	0.50 ± 0.06	0.48 ± 0.04
Cd 228.802 ^a^	0.005	-	-	2.88 ± 0.06	2.82 ± 0.04	1.52 ± 0.03	1.50 ± 0.02
Co 228.616 ^a^	0.005	-	-	0.39 ± 0.03	0.38 ± 0.01	0.58 ± 0.01	0.57 ± 0.02
Cr 267.716 ^a^	0.031	1.69 ± 0.13	1.61 ± 0.09	-	-	1.99 ± 0.03	2.01 ± 0.02
Cu 324.754 ^a^	0.007	7.77 ± 0.53	7.75 ± 0.40	12.2 ± 0.86	12.3 ± 0.03	4.70 ± 0.14	4.80 ± 0.10
K 769.896 ^r^ (%)	0.099	1.91 ± 0.12	1.92 ± 0.10	2.90 ± 0.03	2.92 ±0.1	2.68 ± 0.5	2.72 ± 0.2
Mg 279.553 ^r^ (%)	0.047	0.29 ± 0.02	0.29 ± 0.01	*0.9*	0.91 ± 0.1	*1.20*	1.22 ± 0.3
Mn 257.610 ^a^	0.004	191 ± 12	191 ± 10	76.0 ± 1.2	76 ± 0.02	246 ± 7.1	245 ± 1.5
Mo 202.030 ^a^	0.006	*0.520*	0.49 ± 0.01	-	-	-	-
P 177.495 ^a^ (%)	0.044	*0.25*	0.26 ± 0.01	0.52 ± 0.01	0.51 ± 0.10	0.22 ± 0.01	0.23 ± 0.03
S 180.731 ^a^ (%)	0.088	0.24 ± 0.01	0.25 ± 0.01	*0.50*	0.50 ± 0.05	*0.96*	1.00 ± 0.10
Sr 407.771 ^a^	0.008	37.6 ± 2.7	38.1 ± 0.9	55.5 ± 0.5	54.9 ± 0.08	*85*	89.1 ± 4.5
Yb * 328.937 ^a^	0.010	-					

^a^—axial view; ^r^—radial view; *—internal standard; italic value—information value.

**Table 8:** Instrumental parameters of the F AAS technique with the limits of quantification (LOQ) for Fe, Zn and Pb.

**Measurement** **Parameters**	**Fe 248.814** nm	**Zn 213.857** nm	**Pb 217.000** nm
Gas flow rate [L/h]	60	50	65
LOQ [mg/L]	0.100	0.015	0.067
Flame type	air–acetylene	air–acetylene	air–acetylene

**Table 9:** Results obtained for the studied certified materials using F AAS [mg/kg].

**Analyte**	**INCT-MPH-2** Certified	**INCT-MPH-2** Obtained x_mean_ ± SD	**1570a** Certified	**1570a** Obtained x_mean_ ± SD	**1573a** Certified	**1573a** Obtained x_mean_ ± SD
Fe	*460*	455 ± 5.5	-	-	368 ± 4.3	370 ± 2.5
Pb	2.16 ± 0.23	2.15 ± 0.15	*0.2*	0.19 ± 0.05	-	-
Zn	33.5 ± 2.1	34.2 ± 0.95	82.3 ± 3.9	83.2 ± 2.5	30.9 ± 0.55	40.2 ± 0.35

italic value—information value.

**Table 10:** The operating parameters of the MA-3000 mercury analyser (organism method).

**Stage of Decomposition**	**Heat Temperature** [°C]	**Heat Time** [s]	**Flow** [L/min]	**Slope Time** [s]
Atomize 1	180	120	0.4	120
Atomize 2	850	120	0.4	30

## Data Availability

The data presented in this study are available on request from the corresponding author.
